# Macrophage Depletion via Clodronate Pretreatment Reduces Transgene Expression from AAV Vectors In Vivo

**DOI:** 10.3390/v13102002

**Published:** 2021-10-06

**Authors:** Darrick L. Yu, Natalie S. M. Chow, Byram W. Bridle, Sarah K. Wootton

**Affiliations:** Department of Pathobiology, University of Guelph, Guelph, ON N1G 2W1, Canada; darrickyu@gmail.com (D.L.Y.); nataliesmchow@gmail.com (N.S.M.C.); bbridle@uoguelph.ca (B.W.B.)

**Keywords:** adeno-associated virus, clodronate, macrophage, gene therapy

## Abstract

Adeno-associated virus is a popular gene delivery vehicle for gene therapy studies. A potential roadblock to widespread clinical adoption is the high vector doses required for efficient transduction in vivo, and the potential for subsequent immune responses that may limit prolonged transgene expression. We hypothesized that the depletion of macrophages via systemic delivery of liposome-encapsulated clodronate would improve transgene expression if given prior to systemic AAV vector administration, as has been shown to be the case with adenoviral vectors. Contrary to our expectations, clodronate liposome pretreatment resulted in significantly reduced transgene expression in the liver and heart, but permitted moderate transduction of the white pulp of the spleen. There was a remarkable localization of transgene expression from the red pulp to the center of the white pulp in clodronate-treated mice compared to untreated mice. Similarly, a greater proportion of transgene expression could be observed in the medulla located in the center of the lymph node in mice treated with clodronate-containing liposomes as compared to untreated mice where transgene expression was localized primarily to the cortex. These results underscore the highly significant role that the immune system plays in influencing the distribution and relative numbers of transduced cells in the context of AAV-mediated gene delivery.

## 1. Introduction

Adeno-associated virus (AAV)-mediated gene therapy is approaching a level previously not achieved by any other gene therapy vector. The recent regulatory approval of Luxturna, an AAV gene therapy vector designed to treat patients with a rare form of inherited vision loss [1], and Zolgensma, an AAV gene therapy for type 1 spinal muscular atrophy (SMA) [2], highlights the increasing success such gene therapy strategies are experiencing in the clinic [3]. For continued clinical success, however, there is a need to establish improved vector administration protocols so that maximum transduction of target cells can be achieved while immune responses against the vector itself as well as the transgene product are minimized. In early AAV clinical trials investigating the delivery of factor IX to the liver of hemophilia B patients, it was discovered that although therapeutic levels of factor IX could be achieved at the highest dose of AAV-factor IX, immune responses against the vector capsid limited prolonged expression of the transgene beyond 8 weeks [4]. Previous work has demonstrated that AAV capsids are able to inadvertently package DNA sequences that are entirely devoid of the cis-acting AAV packaging signals, the inverted terminal repeats (ITRs), resulting in the potential for unintended encapsidation of *rep* and *cap* DNA sequences into recombinant vectors [5]. De novo synthesis of capsid proteins after the transduction of host cells might result in the induction of immune responses against the AAV vector and subsequent elimination by the immune system. This severe limitation may be alleviated by using a specialized capsid expression cassette that has an oversized intron, rendering the capsid DNA too large for packaging into an AAV virion [5]. Such strategies may improve the successful transduction of target sites that lie outside of immunoprivileged sites such as the eye. Indeed, AAV trials targeting the eye (subretinal space) have seen more success than tissues more heavily surveyed by the immune system [6]. In a phase 3 trial utilizing an AAV2 vector encoding the hRPE65 required for isomerohydrolase activity of the retinal pigment epithelium, patients demonstrated sustained improvement in both subjective and objective measurements of vision, including improved light sensitivity, visual fields, and functional vision under dim lighting conditions [7], with such benefits lasting for at least three years [8].

In addition to adaptive immunity, innate immune mechanisms might play a role in eliminating much of the administered vector prior to transduction. Marginal zone macrophages play an essential role in eliminating blood-borne pathogens present in circulation as blood filters through the spleen [9,10]. The bacteria *Streptococcus pneumoniae*, *Haemophilus influenzae*, and *Neisseria meningitidis* and viruses adenovirus serotype 5 and cowpox virus may be cleared in this manner [11]. Marginal zone macrophages of the spleen have been shown to accumulate adenovirus serotype 5 in vivo following IV injection [12].

In contrast to the relatively low immunogenicity associated with AAV administration, adenoviral vectors exhibit a much higher degree of adversity when facing the innate and adaptive arms of the immune system [13,14]. AAV can transiently induce the expression of chemokines TNF-alpha, RANTES, IP-10, MIP-1B, MCP-1, and MIP-2 following intravenous administration in mice; however, levels decline to baseline levels 6 h post vector administration [15]. In contrast, adenoviral vectors induced the expression of TNF-alpha and chemokines for over 24 h post administration [15] and induced the expression of inflammatory cytokines in vitro, whereas AAV vectors did not [15]. Several attempts to tame host roadblocks to successful transduction have incorporated the pre-administration of liposome-encapsulated bisphosphonate clodronate in order to deplete phagocytic cells from experimental animals prior to the administration of adenoviral vectors [16,17,18]. Transient depletion of Kupffer cells through intravenous administration of chlodronate liposomes resulted in improved delivery of adenoviral DNA to the liver, prolonged as well as increased transgene expression, and delayed the clearance of vector DNA [18].

Based on the favorable results observed from the studies utilizing adenovirus, we hypothesized that transduction studies with AAV vectors might also benefit with pretreatment using clodronate-containing liposomes. Mice were given clodronate liposomes, and 48 h later, adeno-associated virus vectors expressing a human placental alkaline phosphatase reporter gene. Contrary to previous reports utilizing adenoviral vectors, clodronate liposome pretreatment resulted in a significant decrease in transgene expression in heart and liver tissue (*p* < 0.05), and a possible trend towards a decrease in the spleen.

## 2. Materials and Methods

### 2.1. Cell Culture

Human embryonic kidney (HEK) 293 cells were maintained in Dulbecco’s modified Eagle’s medium supplemented with 10% fetal bovine serum (GIBCO, Invitrogen, Carlsbad, CA, USA), 100 units/mL penicillin, 100 μg/mL streptomycin, and 2 mM l-glutamine in a humidified 5% CO_2_ atmosphere at 37 °C.

### 2.2. AAV Vector Plasmid Construction

Vectors encoding human alkaline phosphatase reporter gene (hPLAP) were derived from an AAV vector plasmid, A_EE_E_1_AP, described previously [19]. The vector was modified to replace the Enzootic-Nasal Tumor Virus-1 (ENTV-1) enhancer/promoter component with enhancer elements derived from Jaagsiekte Sheep Retrovirus (JSRV), acting on the chicken beta actin promoter to drive expression of a human alkaline phosphatase reporter gene (hPLAP). Splicing of the hPLAP encoding transcript was promoted by the presence of a murine leukemia virus polymerase intron found between the enhancer/promoter and the hPLAP gene. Following the hPLAP gene was the SV40 polyA tail for the polyadenylation of transcripts. This enhancer/promoter combination has proven to be effective in driving constitutive expression in a variety of tissues, but is especially active in the lung and liver.

### 2.3. AAV Vector Production and Quantification

The AAV vector was produced as described previously [19]. Briefly, the plasmid containing the AAV genome was co-transfected into cells along with pDGM6, a packaging plasmid that expresses AAV6 capsid. AAV vector titers were determined by quantitative polymerase chain reaction analysis as previously described [20].

### 2.4. AAV Vector Delivery

Mouse experiments were performed in accordance with the guidelines set forth by the Canadian Council on Animal Care (CCAC) and Animal Utilization Protocol #3827 (approved 22 September 2017). Eight-week-old C57BL6/J mice were obtained from Charles River Laboratories (Saint-Constant, QC, Canada). Systemic delivery of AAV vectors was performed by intravenous delivery of a phosphate-buffered saline (PBS) solution containing 1 × 10^11^ vector genomes of AAV vectors injected in a 200 μL volume into the tail vein. Mice were euthanized 1 month post vector administration, and lungs were perfused through the heart with 20 mL of PBS and then separated into individual lobes. For consistency, the same lobe from each mouse was either flash frozen in liquid nitrogen or fixed in 2% paraformaldehyde–PBS for 2.5 h at 22 °C. Half of other major organs, including the liver, spleen, pancreas, lymph node, heart, and kidney, were fixed for 24 h at 22 °C, with the other half placed into liquid nitrogen for subsequent enzymatic assay of hPLAP activity.

### 2.5. Depletion of Macrophages Using Clodronate Liposomes

Clodronate liposomes were prepared as described previously [21]. Forty-eight hours prior to administration of AAV vectors, mice were given a 33 μL injection of clodronate liposomes through the tail vein [22,23]. At this volume, each mouse received 165 µg of liposome-encapsulated clodronate. A 200 μL injection was also given to determine if the effect could be ramped up in a dose-dependent manner.

### 2.6. Quantification of Total Splenocytes in Mice

Splenocytes were enumerated in the following manner: spleens were cut in half, with both halves pressed between the frosted ends of microscope slides to make a single-cell suspension, followed by manual counting using an improved Neubauer hemocytometer. Mean and standard deviation were calculated for PBS and the 33 μL treatment group.

### 2.7. Quantification of Mouse Splenic Macrophages

The method of Rose et al. was used to quantify numbers of marginal zone macrophages [24]. Briefly, spleens were harvested and a cell suspension was made after dicing spleens and treatment with DNase I and collagenase D. 1 × 10^6^ to 2 × 10^6^ cells were pelleted at 1500 rpm for 3 min and supernatant discarded. Cell suspension was incubated with 0.5 µg Fc Block (BD Biosciences, San Jose, USA) for 10 min at RT, followed by a wash with FACS buffer (0.5% BSA in saline). Supernatant was discarded once again and the following antibodies were added to each well: CD45R (B220) clone RA3-6B2 PE-Texas Red (1:400, BD Biosciences), Ly6C clone AL-21 APC-Cy7 (1:500, BD Biosciences), Ly6G clone 1A8 PE (1:400, BD Biosciences), NK1.1 clone PK136 APC (1:300, BD Biosciences), CD11b clone M1/70 FITC (1:160, eBioscience, San Diego, CA, USA), CD11c clone HL3 PE-Cy7 (1:125, BD Biosciences) with 20% 7-AAD in FACS buffer. The stain and cell mixture was incubated for 20 min on ice in the dark, and washed twice in FACS buffer. Lastly, samples were resuspended in FACS buffer and kept on ice, in the dark, until being run on a FACS Aria flow cytometer (BD Biosciences). Mean and standard deviation were calculated for PBS and the 33 μL treatment group, and Student’s t-test was used to determine if differences between these groups was significant.

### 2.8. hPLAP Staining

Tissues were stained for vector-encoded heat-stable hPLAP expression as described previously [19]. Gross pictures of stained tissues were taken using a Zeiss dissecting scope (Zeiss Canada, Toronto, Canada). After fixation tissues were embedded in paraffin wax using an automatic tissue processor, tissue sections (5 μm) were cut and placed on positively charged slides. Since the hPLAP staining intensity is reduced after tissue processing, tissue sections are re-stained to detect hPLAP expression. Tissue sections were deparaffinized and equilibrated in hPLAP buffer (100 mM Tris-HCl pH 8.5, 100 mM NaCl, 50 mM MgCl_2_) for 5 min. Slides were incubated overnight in hPLAP stain (100 mM Tris-HCl pH 8.5, 100 mM NaCl, 50 mM MgCl_2_, 0.34 mg/mL nitroblue tetrazolium salt, 0.17 mg/mL X-phos) on rotator in the dark. After washing twice for 2 min in PBS, tissues were counterstained for 1 min with nuclear fast red solution (Millipore Sigma, Oakville, ON, Canada).

### 2.9. Determination of hPLAP Enzymatic Activity

Mouse tissues were harvested 28 days after AAV vector administration, snap-frozen in liquid nitrogen, and stored at −80 °C until assayed. Tissues were homogenized in TMNC lysis buffer (50 mM Tris HCl pH 7.5, 5 mM MgCl_2_, 100 mM NaCl, 4% (*wt*/*vol*) CHAPS) using a Precellys 24 homogenizer (Bertin Technologies, Montigny-le-Bretonneux, France) with ~200 μL of TMNC buffer in a FastPrep™ Lysing Matrix A tube (MP Bio, Santa Ana, CA, USA). Tissue homogenates were placed in a water bath at 65 °C for 1 h to inactivate endogenous heat-labile AP activity and subsequently clarified by centrifugation at 17,900× *g* for 15 min at 4°C to remove cell debris. The protein content of each sample was determined by the method of Bradford, and the AP activity in tissue lysates was determined by a fluorometric assay using the 4-methylumbelliferyl phosphate (MUP) (Sigma, St. Louis, MO, USA) substrate, as described previously [19]. The mean and standard deviation were calculated for AP activity for treated and non-treated groups and in each of the organs: lung, liver, heart, and spleen. Student’s *t*-test (unpaired, two-tailed) was used to determine if differences between clodronate-treated and untreated groups were significant.

## 3. Results

### 3.1. Absolute Splenocyte Quantification by Manual Counting and Marginal Zone Macrophage Quantification by Flow Cytometry Following Clodronate Liposome Pretreatment

**Figure 1:**
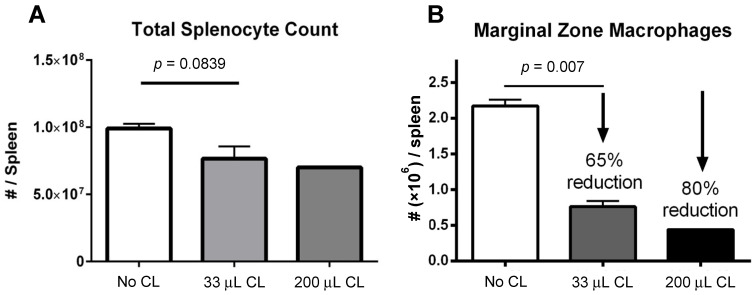
(**A**) Manual count of total numbers of splenocytes following treatment of mice with clodronate liposome treatment (CL) at two different volumes (*n* = 4 per group). There appeared to be a greater decline in total splenocyte numbers with the higher dose of clodronate liposomes, but this was not statistically significant. (**B**) Marginal zone macrophage count as assessed by flow cytometry following clodronate liposome treatment. Treatment with 33 μL of CL yielded a 65% reduction in numbers of marginal zone macrophages. This difference was statistically significant (*p* < 0.05). With a higher dose of 200 μL, marginal zone macrophage numbers could be reduced even further (80%).

A decrease in absolute splenocyte numbers was observed for mice given two different doses (33 and 200 μL) of clodronate liposomes (Figure 1A). A trend towards decreasing numbers of total splenocytes was observed in mice given clodronate compared to control mice. Flow cytometric analysis revealed a significant decrease in marginal zone macrophage number was observed following clodronate pretreatment (*p* < 0.007) (Figure 1B). In particular, there was an observed 65% decline in the numbers of marginal zone macrophages following a 33 μL intravenous administration of clodronate liposomes through the tail vein. Increasing the dose to 200 μL resulted in an 80% reduction in marginal zone macrophages; however, increasing the dose to this level may have also affected other cell types as well. 

### 3.2. Gross Analysis of Human Placental Alkaline Phosphatase (hPLAP) Transgene Expression in Various Tissues

**Figure 2:**
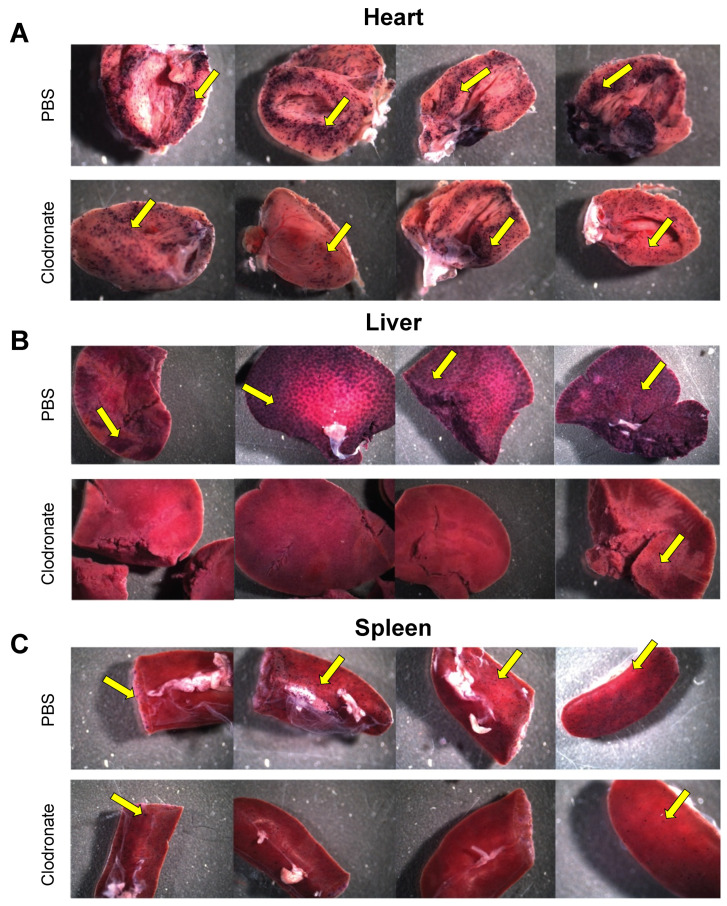
Gross comparison of mice (*n* = 4) transduced systemically (via tail vein) with 1 × 10^11^ vg of AAV6 vector expressing human placental alkaline phosphatase (hPLAP) after being given an intravenous injection of clodronate liposomes or PBS, 48 h prior. Mice were euthanized 28 days post-AAV administration and tissues fixed and stained for hPLAP expression. There appeared to be a trend towards more expression in clodronate-untreated mice, particularly in the heart (**A**) and liver (**B**), but less so in the spleen (**C**). Images pictured are from individual mice. Yellow arrows point to regions of purple punctate staining representative of hPLAP expression.

**Figure 3:**
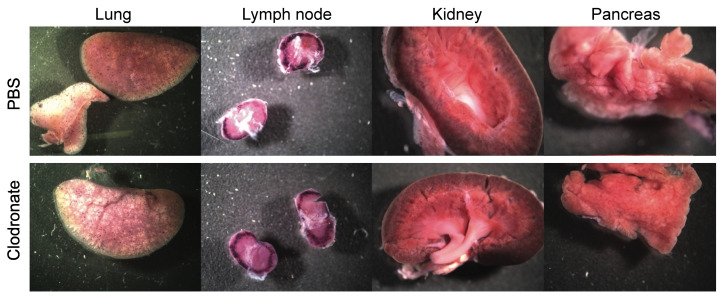
Representative gross images of lung, lymph node, spleen, and kidney from clodronate-treated mice and control mice. Both groups (*n* = 4) were administered 1 × 10^11^ vg of AAV vector expressing human placental alkaline phosphatase (hPLAP) via tail vein injection and euthanized 28 days later. Tissues were fixed and stained for hPLAP expression. There did not appear to be grossly visible differences between these two groups of mice in these particular organs. Expression of hPLAP in these organs was relatively low.

hPLAP reporter gene expression, as evidenced by purple staining, was observed in a variety of tissues 28 days following intravenous delivery of 1 × 10^11^ vg of an AAV6 vector expressing hPLAP with and without prior clodronate treatment. Most notably, expression appeared to be lower in clodronate-treated tissues than in non-treated controls (Figure 2). This was especially evident in the tissues of the heart (Figure 2A) and liver (Figure 2B). Following intravenous AAV administration, the expression of the reporter gene was restricted to particular patches in the lungs of both clodronate-treated and non-treated mice, likely owing to the architecture of the lungs and the fact that intravenously administered vector could access the lungs only at pulmonary or bronchial blood vessels (Figure 3). However, in terms of improved expression, overall, the trend appeared to favor untreated animals.

### 3.3. Quantification of hPLAP Expression in Various Tissues

**Figure 4:**
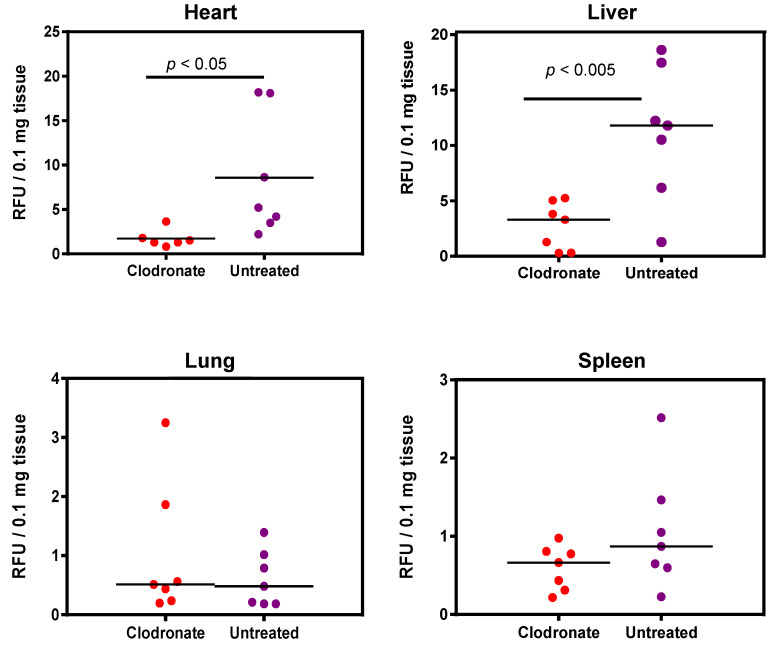
Quantification of alkaline phosphatase activity following systemic administration of 1 × 10^11^ vg of an AAV6 vector expressing human placental alkaline phosphatase (hPLAP) with and without prior intravenous administration of clodronate. In the heart (*p* < 0.05) and liver (*p* < 0.005), a statistically significant difference could be observed in clodronate-treated and untreated mice, with untreated mice possessing on average ~4 fold more alkaline phosphatase activity. No significant difference could be observed in the levels of alkaline phosphatase activity in mouse lung and spleen.

Enzymatic assay quantification of hPLAP was conducted after the homogenization of treated and untreated mouse tissues, as described previously [19]. Mean and standard deviation were calculated for treated and untreated groups for lung, liver, heart, and spleen, and Student’s *t*-test (unpaired, two-tailed) was used to determine if differences were significant (*p* < 0.05). Pooled data from two independent experiments (*n* = 3 and *n* = 4 for the first and second experiment, respectively) demonstrated a significant decrease in expression for clodronate-treated mice in the liver and heart compared to untreated mice (*p* < 0.005 and *p* < 0.05, respectively). No significant trend was observed in the lungs, probably due to the low level of expression in this organ. A possible statistical trend towards a decrease in transgene expression was observed in the spleen (*p* < 0.1) (Figure 4).

### 3.4. Histological Analysis of hPLAP Transgene Expression in Clodronate-Treated Mice and PBS-Treated Controls

**Figure 5:**
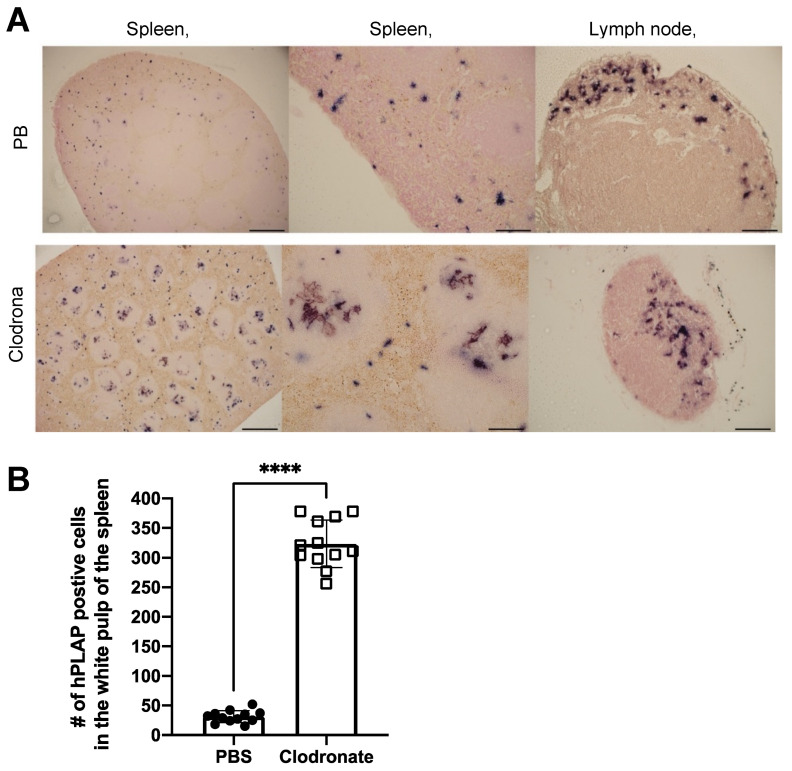
Histochemical staining of sections from spleen and lymph node in clodronate-treated and control mice. (**A**) Representative tissue sections of spleen and lymph node from clodronate-treated mice and control mice. Both groups were administered 1 × 10^11^ vg of AAV vector expressing human placental alkaline phosphatase (hPLAP) via tail vein injection and euthanized 28 days later. Tissues were formalin fixed, paraffin embedded, and stained for hPLAP expression. Administration of clodronate appeared to allow improved AAV transduction of white pulp relative to control mice. hPLAP staining in the center of the white pulp could be observed for clodronate-treated mice, whereas with control mice, no such staining could be observed. The presence of marginal zone macrophages likely prevented efficient transduction of white pulp in control mice. Within the lymph node, staining seemed to be restricted to the periphery of the lymph node for control mice, whereas in clodronate-treated mice, staining could be observed in the center. (**B**) Quantification of hPLAP positive foci in the white pulp of spleens from clodronate-treated and control mice. Foci of hPLAP stained cells in the white pulp of the spleen from three tissue sections (4×) per mouse per group were counted. 4× scale bar = 200 μM, 10× scale bar = 100 μM, 20× scale bar = 50 μM. Data were analyzed using an unpaired *t*-test, **** = *p* <0.0001.

Tissues of untreated mice and mice treated with clodronate were paraffin embedded, sectioned, and viewed under a light microscope. Differences were observed in the pattern of expression of hPLAP within the spleens of treated and untreated mice (Figure 5). A greater proportion of hPLAP expression was observed within the center of white pulp in clodronate-treated mice compared to untreated mice. Due to the decline in marginal zone macrophage numbers because of clodronate treatment, a greater proportion of the vector may have been able to penetrate into the white pulp and persist long enough to transduce cells within the white pulp as compared to the non-treated mice. The white pulp of the spleen is a lymph node-like organ composed of lymphoid sheaths with both B and T cell compartments surrounding a central arteriole [25]. Interestingly, marginal zone B cells can become potent antigen presenting cells after uptake of soluble antigen [25]. Dendritic cells can also migrate into the white pulp following activation [25]. The dark alkaline phosphatase staining observed in the white pulp of clodronate-treated mice may have been antigen presenting cells that had been transduced by the vector and subsequently migrated into the white pulp following activation. A similar pattern of expression was observed for the lymph node, where a greater proportion of expression could be observed in the medulla located in the center of the lymph node.

**Figure 6:**
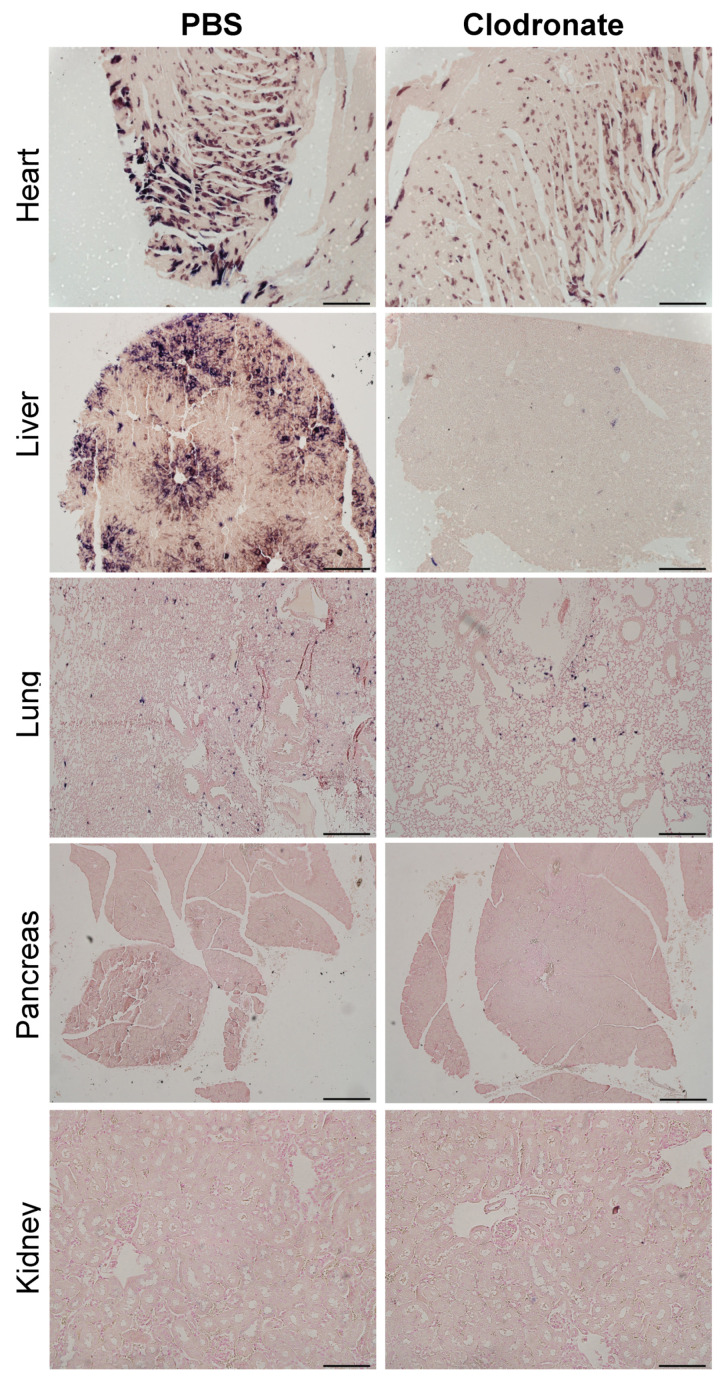
Representative tissue sections of the heart, liver, lung, and pancreas from clodronate-treated and control mice (PBS). Both groups were administered 1 × 10^11^ vg of AAV vector expressing human placental alkaline phosphatase (hPLAP) via tail vein injection and euthanized 28 days later. Tissues were formalin fixed, paraffin embedded, and stained for hPLAP expression. Images were taken at 10× magnification for all tissues except the pancreas, which was imaged at 4×. 4× scale bar = 200 μM, 10× scale bar = 100 μM.

As was observed grossly, there was a marked reduction in transgene expression in tissues sections from the heart and liver of clodronate-treated mice, whereas there was no obvious difference in hPLAP staining in lung sections from clodronate-treated mice compared to PBS controls (Figure 6). Little to no transgene expression was observed in tissue sections from the kidney and pancreas in either treatment group. In tissues where transgene expression was detected, there did not appear to be a difference in the cell types that were transduced.

## 4. Discussion

In contrast to the improvements in transgene expression observed with adenoviral vectors, pretreatment with clodronate liposomes did not appear to improve AAV-mediated gene delivery, and may have actually had the opposite effect. Significantly reduced expression was observed in mice that had been pretreated with clodronate, particularly in the heart and liver. Little or no difference was observed in transgene levels in the lungs between treated and non-treated groups. This might be attributed to the fact that the transduction of the lungs was poor due to the intravenous route of administration of the AAV vector, resulting in only minute patches of transduction where the vasculature is able to access lung tissue. However, in all other tissues assayed, transduction was lower in the clodronate-treated group. It is unclear why transduction might be lower in these tissues; however, several theories are postulated. Intravenous injection of clodronate liposomes ensures that all phagocytes having direct access to the blood can potentially engulf clodronate liposomes [26]. While clodronate-containing liposomes can deplete all professional phagocytes, including both macrophages and dendritic cells, the liver and spleen are so efficient at removing foreign particles from the bloodstream that the exposure of phagocytes outside of these organs to blood-borne clodronate liposomes would be limited [27]. Particular macrophage populations eliminated by clodronate treatment may have a role in tolerizing the host to components of the vector or the transgene itself. For example, the liver has been known to participate in the induction of tolerance to foreign antigens [28]. Breous and colleagues determined that resident liver macrophages, known as Kupffer cells, can work in conjunction with hepatic regulatory T cells to create a local immunosuppressive environment that inhibits the establishment of a cytotoxic T lymphocyte (CTL) response [29]. Specifically, they found that the elimination of the Kupffer cell population using clodronate was able to completely abolish the expression of the immunosuppressive cytokine IL-10. Furthermore, primary administration of an AAV vector encoding human alpha-1 anti-trypsin (hAAT) enabled systemic tolerance to a secondary administration of an adenoviral vector also encoding hAAT, resulting in greatly improved expression compared to the adenovirus vector alone. The induction of tolerance was shown to be specific to hAAT, as transduction with an Ad-LacZ vector after prior AAV-hAAT administration elicited a strong CTL response [29]. To the best of our knowledge, there are no reports in the literature of macrophages facilitating the distribution of AAV particles to other target tissues, although it remains a possibility. However, it is well known that tissue macrophages engulf AAV non-specifically by phagocytosis, process the capsid, and following migration to a draining lymph node, present capsid-derived peptides to effector lymphocytes [30].

The results demonstrated herein and in prior publications underscore the highly significant role that the immune system plays in sculpting the distribution and relative numbers of transduced cells in the context of AAV-mediated gene delivery. It appears that the phagocytic activity of macrophages is greatly outweighed by the contributions of adaptive immunity, at least for prolonged periods of transgene expression matching or exceeding four weeks. The inhibition of phagocytic uptake by macrophage depletion via clodronate liposomes may, however, be beneficial when a transient, limited period of expression is desired. Initial transduction may be greater under these circumstances due to decreased numbers of phagocytic cells capable of ingesting vector particles, but would likely decrease over time as an immune response against the transgene is mounted. Future experiments will evaluate AAV-mediated transgene expression in clodronate liposome-treated and untreated mice at earlier time points (e.g., 10–14 days post-transduction) so as to allow for the quantification of transgene expression prior to the onset of the host immune response to the therapy. Additionally, we would like to investigate the effect of administering clodronate liposomes via the intranasal route to specifically deplete alveolar macrophages [31], which might allow for greater lung specific transduction while leaving Kupffer cells intact.

In contrast to the results presented here, gene delivery studies by Wolff and colleagues employing adenovirus resulted in a marked increase in transgene expression when clodronate pretreatment occurred in advance of adenovirus transduction [18]. This difference may be attributed to differences in the biology of adenovirus and adeno-associated virus. Whereas adenovirus is highly immunogenic, inducing the expression of inflammatory cytokines for prolonged periods of time upon cell transduction, AAV is far less so [15]. Therefore, the innately high immunogenicity of adenovirus may make it more difficult to induce tolerance to these vectors, and therefore it might not occur as readily as with adeno-associated virus. Thus, the inhibition of phagocytosis might be a relatively less important process than removing phagocytic cells prior to administration, within the particular context of adenovirus vectors. In addition, differences in vector titers at the point of administration might account for differences in transgene expression between Ad and AAV vectors. Whereas in our study an AAV vector dose of 1 × 10^11^ vector genomes was delivered intravenously, Wolff et al. administered either 5 × 10^6^ or 1 × 10^7^ PFU [18]. Comparisons between genome copy number and plaque forming units might be fraught with difficulty; however, if taken at face value, the number of vector particles was greater in favor of AAV by a factor of 10,000. This number also does not account for the presence of empty AAV capsid particles, which capsids can vary from 10% to 90% [32]. Empty particles might therefore act in a “blocking” fashion, possibly overwhelming the phagocytic capacity of macrophages and thereby making the transient elimination of phagocytic cells unnecessary for AAV transduction [22]. In another study by Aalbers et al., intravenous administration of clodronate liposomes 48 h prior to intra-articular AAV5 vector administration improved transgene expression [22]. One reason for the discrepancy between their findings and ours may have to do with how AAV was administered. AAV was administered systemically in our study, whereas AAV was administered locally to the knee joint in the study by Aalbers et al., thereby limiting the exposure of AAV to synovial macrophages.

Future gene therapy protocols may seek to increase the number of Kupffer cells rather than eliminate them, or to promote the interaction of Kupffer cells with hepatic regulatory T cells with the aim of boosting tolerance [29]. Improved protocols might also incorporate targeted transduction of the liver in order to increase the likelihood of the induction of tolerance, even if the target tissue is distal to the liver. With increased immune tolerance, a reduction in the number of vector particles required for efficient transduction might be possible, leading to the decreased presentation of vector-associated antigens and opening the possibility of repeated vector administrations using the same capsid. A reduction in the number of vector particles required for successful transduction in large animals such as humans would likewise also be a welcome change, as the production of the vast quantities of AAV vectors needed for clinical use is difficult and costly.

## Data Availability

Raw data were generated at the University of Guelph. Derived data supporting the findings of this study are available from the corresponding author on request.
